# Annotations

**Published:** 1911-03-11

**Authors:** 


### ANNOTATIONS.

### The Smallpox Outbreak.

It is nearly ten years since smallpox invaded London in epidemic proportions, and meanwhile the vaccination laws have been steadily more and more relaxed by invertebrate Ministers (of both political parties) under pressure from the anti-vaccinationists. Some day, beyond doubt, London and the kingdom in general will have to pay dearly for the wilful blindness of those whom the electorate chooses to govern them. The writing on the wall is clear enough, if only the people would take the trouble to read it. In the present case it seems as if the outbreak is being successfully limited and controlled, though at enormous labour and expense in tracing contacts; but this is all the more reason why some intelligent attempt should be made to prevent altogether these epidemics, which are fraught with such tremendous possibilities of disaster to the nation. We have only to look across the North Sea for an example in this, as in so many other questions of common-sense government. There is to be noted the most scientific system of smallpox eradication so far evolved; it is, of course, based upon compulsory vaccination and re-vaccination. They have no use for the "anti" and the faddist in Germany; and the result is that there are fewer cases of smallpox annually in Germany than there have been already in London alone this past month. Germany is well known to be the best vaccinated country in the world, and also the least invaded by smallpox; and this notwithstanding her extensive land frontier with Russia. This simple fact ought to be enough to convince anyone who is open to argument; but if more is needed, it is to be observed how the statistics of the present outbreak support the experience derived from that of previous epidemics. The very latest statistics to date are not yet available at the time of going to press; but at the end of last week it appeared that of the patients under eight years of age *not one had ever been vaccinated*; of those from that age upwards a good many had been vaccinated in infancy, but *not a single one had ever been re-vaccinated*. Further, no deaths had up to then taken place among the vaccinated, though two or three had been notified among the others.

### The Notification of Leprosy.

It is now some centuries since the strict enforcement of segregation abolished leprosy as one of the endemic diseases of Britain. Ever since the Middle Ages, a case of leprosy is with us a medical curiosity, imported always from abroad. In a word, we have no leper problem in the same sense that they have it in Norway, Spain, Africa, Peru, Paraguay, and other parts of the world. What, then, is the legal status of a leper who comes to this country after contracting the disease? The question has been discussed by the members of the Laryngological Section of the Royal Society of Medicine in connection with a case of unquestioned leprosy, with nasal and laryngeal lesions exhibited, by Dr. H. J. Davis. In this particular instance the patient was under care at the West London Hospital, where, of course, due precautions would be taken to avoid any spread of contagion. But Dr. Sinclair Thomson was moved to the recollection of a coloured patient whom he exhibited two years ago. This man went openly about his avocations without restraint, to quote the words of the report in the *Journal of Laryngology*. The speaker pointed out that leprosy is not a notifiable disease in this country, a fact which is certainly most extraordinary. Dr. Dan McKenzie followed with an account of a patient of his own, who suffered from the disease and who was employed in business in London until shortly before his death. It may be that the old leper laws are still on the Statute Book, and could be invoked to deal with cases of this sort; but decidedly it would be less disquieting if leprosy were made a notifiable disease and brought into line with such infective disorders as scarlet fever and the like, under the control of the local sanitary authorities. Without raising a scare, it is incumbent on the profession to point out to the public and the legislature that absolutely no precautions are at present taken to prevent a leper from disseminating his disease in this country, a state of things which can only be described as most unsatisfactory.

### Surgical "Negligence" in France.

Following close upon the *cause célèbre* of a month ago, in which a London surgeon was sued for alleged negligence in the performance of an operation, is a case of a similar type in France. The details appear to be rather different, in that it was established that two swabs were unintentionally left behind; whereas in the London case it was shown that the swab was purposely inserted for a definite and legitimate object. At any rate, the French surgeon has been mulcted in damages to the sum of £200; and this result has drawn from the eminent French medical jurist an elaborate protest against both the legality and the morality of the decision. He argues that in the flurry of a long and difficult operation, done in an emergency, such as for perforated gastric ulcer or similar crisis, there is no blame to be attached to the surgeon who occasionally overlooks a sponge. As well, he argues, penalise a fireman who sprains the wrist of a girl he is rescuing from the top window of a blazing house. In Britain at least it is improbable that this argument will produce any effect. No greater mistake can be imagined that to absolve surgeons of the responsibility for counting their swabs and their instruments; though at the same time there should be adequate checks against the bringing of speculative, baseless, and blackmailing actions such as that which recently failed in our courts. A well-known line of mail steamers has a rigid rule by which every captain whose ship touches ground is at once dismissed, no matter whose fault the accident may be. The rule occasionally works intense individual hardship; but it makes for the protection of the travelling public without a doubt. So it is also with the surgeon and his swabs, and quite rightly.

